# Effect of Surfactant Formula on the Film Forming Capacity, Wettability, and Preservation Properties of Electrically Sprayed Sodium Alginate Coats

**DOI:** 10.3390/foods12112197

**Published:** 2023-05-30

**Authors:** Wanqing Deng, Huiyuan Zheng, Zichun Zhu, Yun Deng, Yuchen Shi, Danfeng Wang, Yu Zhong

**Affiliations:** 1 Department of Food Science & Technology, Shanghai Jiao Tong University, Shanghai 200240, China; dwqdwq9218@163.com (W.D.); 369402243@sjtu.edu.cn (H.Z.); zhizichunegghead@sjtu.edu.cn (Z.Z.); y_deng@sjtu.edu.cn (Y.D.); llz26@sjtu.edu.cn (D.W.); 2 Shanghai SOLON Information Technology Co., Ltd., Shanghai 201108, China; yshi8624@gmail.com

**Keywords:** sodium alginate, hydrophile-lipophile balance (HLB), edible coatings, blueberry

## Abstract

Surfactants are always added to coating formulations to ensure good adhesion of edible coatings to a product’s surface and to maintain freshness. In this study, the effects of the mix surfactants Tween 20 and Span 80 with different hydrophile–lipophile balance (HLB) values on the film-forming ability, wettability, and preservation capacity of blueberry sodium alginate coating were investigated. The results indicated that Tween 20 obviously ensured favorable wettability and improved the uniformity and mechanical properties of the resulting film. While the addition of Span 80 reduced the mean particle size of the coating, enhanced the water resistance of the film, and helped to reduce blueberry weight loss. A sodium alginate coating with low viscosity and medium HLB could better inhibit the galactose, sucrose, and linoleic acid metabolism of blueberries, reduce the consumption of phenols, promote the accumulation of flavonoids, and thus display superior coating performance. In summary, sodium alginate coating with medium HLB had comprehensive advantages in film-forming ability and wettability and was conducive to the fresh-keeping role.

## 1. Introduction

Blueberries are one of the five healthy foods stipulated by the Food and Agriculture Organization of the United Nations. They are rich in ascorbic acid, polyphenols, flavonoids, and anthocyanins and have excellent nutritional and healthcare value. However, blueberries are susceptible to biological, physical, and chemical damage during growth and postharvest storage, which seriously affects their quality and safety [1]. Edible coatings can form physical barriers on the surface of fruits and vegetables to reduce mechanical damage, inhibit the growth of microorganisms, decrease physiological activities, and effectively extend the storage period of blueberries [2,3]. Ren et al. [4] prepared zein/chitosan active films incorporated with curcumin and eugenol, which could delay the deterioration of blueberries and extend their shelf life. Tang et al. [5] used aloe vera polysaccharide, β-hydroxy-β-methylbutyrate calcium, and nanocellulose to prepare a composite film, which showed splendid performance in terms of the hardness, respiration rate, weight loss, total soluble solids, and titratable acidity of blueberries.

Among the natural raw materials, sodium alginate is one of the most promising biopolymers due to its low cost, excellent film-forming ability, good water-retaining properties, safety, and stability; it is also widely used for food preservation [6,7]. In the study of Mannozzi et al. [8], it was found that blueberries wrapped with a sodium alginate coating or a sodium alginate plus pectin coating could improve the firmness and apparently reduce the growth kinetics of mesophilic aerobic bacteria and yeasts. The physical properties of sodium alginate varied depending on the chemical compositions such as the proportion of guluronate (G) and mannuronate (M), the intrinsic viscosity (related to molecular mass), the sequential order of these residues, the length of G-blocks, etc., which was essential to developing proper coatings [9]. Marcos et al. [9] found that films prepared from sodium alginate with a high intrinsic viscosity (ƞ-high) showed significantly higher mechanical strength than ƞ-low films; gelation time affected the mechanical properties of ƞ-low films but had no impact on ƞ-high films. In addition, the coating method can also influence the film-forming performance and its subsequent preservation effects. It was reported that electrostatic spray improved the transfer efficiency and uniformity of sodium alginate-based coatings and could delay the decay of strawberries for about 3 days [10].

In order to help the aqueous coatings spread more easily on the hydrophobic surface of fruits and vegetables, surfactants are always added to the coating system. Surfactants could form interactions with the raw materials and had great influences on the intrinsic performances of the film-forming solutions, the microstructures, and the macroscopic properties of the resulting films [11]. It was found that there were electrostatic interactions, hydrophobic interactions, hydrogen bonding interactions, steric hindrance effects, and other interactions between polysaccharide macromolecules and surfactants, which can cause various changes in the critical micellar concentration (CMC) of surfactants, surface adsorption, and interface rheology of the system [12]. Meanwhile, the addition of ionic and nonionic surfactants could help obtain stable systems, and the stability depended on the density, size of particles, pH, ionic strength, and so on [13]. Patrick et al. [14] found that adding a cationic surfactant to a negatively charged sodium alginate coating would greatly impact the electrical properties of the solution, resulting in changes in particle size and zeta potential. Moreover, when the addition of surfactant exceeded CMC, a multilayer-superposition structure between surfactant and substrate was found on the surface of the solution [15]. Nonionic surfactants have low critical micellar concentrations, controllable hydrophilic and hydrophobic properties, and good stabilities, and are frequently applied in the food industry, among which Tween and Span are the two most typical series. Zhong and Li [15] proved that Tween 20 and Span 80 reduced the surface tension and changed the wettability of the kudzu (*Pueraria lobata*) starch film-forming solution. The use of surfactants increased the roughness of the film layer and exhibited a slow-release effect of ascorbic acid.

It is noteworthy that the surfactant ratio, characterized by the hydrophile-lipophile balance (HLB) value, also had a great influence on the film-forming performance. Previous research has established that surfactants with low HLB values could enhance the interactions between substrates and lipids and then improve the distribution uniformity of lipids in films, while those with high HLB values help make the internal structure of films tight [16]. Moreover, recent evidence has shown that the mixed emulsifier of Span 80 and Tween 60 with an HLB value of 11.19 had the strongest effect on improving the emulsion stability [17]. Research conducted by Miele et al. [18] found that sodium caseinate-based coatings with Tween 80 and Span 80 at an HLB of 9.2 had the most physically stable state and reduced the respiration and transpiration rates of strawberries by 17% and 40%, respectively, at 4 °C.

In this study, sodium alginate coating systems composed of two surfactants (Span 80/Tween 20) were prepared and electrostatically sprayed on blueberries. Then the effects of the surfactant ratios with different HLB values on film-forming characteristics and preservation performances were assessed, which shed new light on the influence of surfactants on coating systems.

## 2. Materials and Methods

### 2.1. Materials

Blueberries were obtained from Yunnan Meilong Town Agricultural Technology Co. Ltd. (Wenshan, China). Two kinds of sodium alginate with different molecular weights (cP 80–120, 179 kDa; cP 500–600, 291.1 kDa; analytical grade, purity greater than 90%) were purchased from Fujifilm and Hikaru Chunyao (Shanghai) Chemical Co., Ltd. (Shanghai, China). Methanol, chromatographically pure, was purchased from Shanghai Aladdin Biochemical Technology Co., Ltd. (Shanghai, China). Formic acid and acetonitrile, chromatographically pure, were purchased from Thermo Fisher Scientific (Shanghai, China) Co., Ltd. (Shanghai, China). Other chemical reagents were all of analytical grade and obtained from Sinopharm Group Chemical Reagent Co., Ltd. (Shanghai, China).

### 2.2. Preparation of Sodium Alginate Film

Two kinds of sodium alginate were dissolved in deionized water (18.25 MΩ·cm, the same below), respectively, to prepare a solution with a mass fraction of 0.5% (marked as H and L), into which glycerin of 30% (*w*/*w*) as a plasticizer and 0.05% (*w*/*v*) Tween 20–Span 80 mixture as a surfactant were added. According to the pre-experiments, Tween 20/Span 80 at the ratio of 10:0, 8:2, 4:6, 0:10, with HLB of 16.70, 14.22, 9.26, and 4.30 were selected, and each group was labeled as L_tw_, L_82_, L_46_, L_sp_, H_tw_, H_82_, H_46_, and H_sp,_ respectively. Letter L represents low viscosity sodium alginate, letter H represents high viscosity sodium alginate, and subscripts represent different ratios of Tween and Span (“tw”, “82”, “46”, and “sp” stand for Tween: Span = 10: 0, 8: 2, 4: 6, and 0: 10, respectively). The solution was stirred on a magnetic agitator (C-MAG HS10, Germany; IKA Instrument (Guangzhou) Co., Ltd., Guangzhou, China) until fully dissolved, then treated with an ultrasonic machine (S10H, Zhiwei (Xiamen) Instrument Co., Ltd., Xiamen, China) in a water bath at 60 °C for 45 min, and homogenized at 20,000 rpm for 5 min. The solution was stood at room temperature for cooling and defoaming, and 500 mL of defoamed solution was poured on a horizontal glass plate (25 cm × 25 cm) to obtain the film with a thickness of about 0.04 μm. The film was placed at 25 ± 1 °C and a relative humidity of 60 ± 2% before testing.

### 2.3. Surface Tension of Sodium Alginate Solution

An automatic surface tensimeter (K100, Kruss, Hamburg, Germany) equipped with a platinum ring was used to measure the surface tension of a 20 mL sodium alginate solution at room temperature by the hanging plate method [11]. The type of hanging plate was PL01, and the size parameter was 10 mm × 19.9 mm × 0.2 mm.

### 2.4. Mean Particle Size of Sodium Alginate Solution

The mean particle size of the sodium alginate solution was measured by a laser particle size analyzer (MasterSizer 3000, Malvern Instruments Ltd., Malvern, PA, USA). The results were expressed as D_43_.

### 2.5. Wettability of Sodium Alginate Solution

The dynamic surface tension of the sodium alginate solution and the contact angle on the blueberry surface were measured using a contact angle measuring instrument (SL200KS, Shanghai Solon Information Technology Co., Ltd., Shanghai, China). The surface of the blueberry was cleaned with distilled water, and a piece of epidermis was placed on the slide. The sodium alginate solution was injected into the sampling system. The pendant drop method [11] was used to test the dynamic surface tension, and the Asha algorithm was used to calculate the value. After setting the opening time of the injection valve, 13 droplets of the solution were sprayed on three different locations on the epidermis using the sessile drop method [11], and the contact angles were calculated by the Asha algorithm. Before changing each sample, the liquid injection system was cleaned with distilled water to ensure that the liquid feeder, injection valve, and injection needle were clean.

### 2.6. Mechanical Properties of Sodium Alginate Film

The films were cut into rectangular strips (80 mm × 60 mm), and the tensile strength (TS, MPa) and elongation at break (EAB, %) were measured by a texture analyzer (TA.XTplus, Stable Micro System Ltd., London, UK) according to the method of Zhuang et al. [19]. The initial distance and testing speed were set at 40 mm and 0.2 mm/s, respectively.

### 2.7. Water Vapor Permeability (WVP) of Sodium Alginate Film

The WVP was tested by the cup method, according to Lupina et al. [20]. A permeable cup with an inner diameter of 57 mm and an internal depth of 15 mm was used as the measuring device. Anhydrous calcium chloride was added to the cup and sealed with a film sample of 80 mm × 80 mm. The sample was placed in an environment with a temperature of 25 ± 1 °C and a relative humidity of 60 ± 2%. The WVP (g·mm·m^−2^·d^−1^·Pa^−1^·10^−6^) was calculated by Equation (1).

(1) $$WVP=\Delta m\cdot d/\left(A\cdot \Delta t\cdot \Delta p\right)$$

where Δm is the weight change of water permeated through the film before and after measurement, d is the thickness of the film, A is the water vapor transmission area, Δt is the measurement duration, and Δp is the water vapor pressure difference on both sides of the film.

### 2.8. Microstructure of Sodium Alginate Film

The freeze-dried sodium alginate film was quenched by liquid nitrogen, and the cross-section was observed by SEM (VEGA 3, TESCAN Ltd., Brno, Czech Republic) after vacuum platinization with a high vacuum coating apparatus (Q 150T ES plus, Quorum, East Sussex, UK) for 15 s.

### 2.9. Blueberry Electrostatic Spraying Treatment

Fresh blueberries were washed with deionized water and then carefully wiped dry with a napkin. A sodium alginate solution was coated on blueberries by an electrostatic spraying system (SC-ET, Electrostatic Spraying Systems, Inc., Watkinsville, GA, USA). The spray gun was fixed vertically 40 cm above the blueberry, and the sodium alginate solution with Tween 20/Span 80 at a ratio of 8:2, 4:6, 0:10 was sprayed for 30 s twice on blueberries. The spraying velocity, feed pressure, voltage, and load current were 3.8 L/h, 1.8 kg/cm^3^, 7.5 kV, and 60 mA, respectively. The coated blueberries were fully air-dried and refrigerated at 4 °C, and the quality indexes were measured on days 0, 3, 6, 9, and 15.

### 2.10. Measurement of Storage Quality of Blueberry

#### 2.10.1. Weight Loss

Briefly, 50 blueberries were transferred to the pre-weighed crisper. The mass of the crisper and blueberries was weighed at each time point, and the weight loss (%) was calculated according to Equation (2).

(2) $$\mathrm{Weight}\mathrm{loss}\mathrm{rate}=\left(m_{1}-m_{n}\right)/\left(m_{1}-m_{0}\right)$$

where $m_{0}$ is the weight of the crisper, $m_{1}$ is the total mass of blueberries and crisper on day 0, and $m_{n}$ is the total mass of blueberries and crisper on day n.

#### 2.10.2. Firmness

The texture analyzer (TA.XTplus, Stable Micro System Ltd., London, UK) was used to measure the hardness of blueberries using a P/50 probe. The test speed was 1 mm/s, and the extrusion response variable was 75%.

#### 2.10.3. Apparent Surface Color

A bench colorimeter (Lab Scan XE, Hunter Associates Laboratory, Inc., Fairfax, VA, USA) was used for testing apparent surface color. The light source condition was D65/10, and the results were expressed as a color difference ΔE.

#### 2.10.4. Preparation of Blueberry Extract

Briefly, 10 g of blueberries was added to 25 mL pre-cooled deionized water, pounded with a grinding pestle, homogenized at 20,000 rpm for 60 s using a handheld homogenizer (S10, Shanghai Weimi Technology Co., Ltd., Shanghai, China), and then ultrasonically extracted at room temperature for 1 h. The extract was centrifuged at 4 °C and 8000× *g* for 10 min by a high-speed centrifuge (Z236K, HERMLE (Shanghai) Instrument Technology Co., LTD, Shanghai, China). The supernatant was blueberry water extract, which was transferred and stored for testing. The extraction solution was changed to 1% hydrochloric acid methanol (hydrochloric acid: deionized water: methanol = 1:19:80), and the other steps were the same as water extraction to obtain blueberry methanol extract.

#### 2.10.5. Titratable Acidity (TA)

Briefly, 1 mL of blueberry water extract was diluted 50 times, added with 2~3 drops of 0.5% phenolphthalide solution, and titrated with 0.01M sodium hydroxide solution. Deionized water was used as a blank control, and TA (% crystalline citric acid) was calculated according to Equation (3).

(3) $$TA=c\cdot \left(V_{1}-V_{0}\right)\cdot k\cdot V\cdot m^{-1}$$

where c is the concentration of sodium hydroxide solution, c is the volume of sodium hydroxide consumed by titration, $V_{0}$ is the volume of sodium hydroxide consumed by blank experiment, k is the conversion coefficient of crystalline citric acid (k = 0.07), V is the total volume of water extract, and m is the total mass of blueberry consumed by water extract.

#### 2.10.6. Total Soluble Solids (TSS)

Five blueberries were ground in a mortar until homogenized and then squeezed with gauze to obtain a filtrate. The TSS content of the filtrate was measured by a handheld refraction instrument (Master-M, Japan Aiyan Co. Ltd., Tokyo, Japan), and the results were expressed as %.

#### 2.10.7. Total Phenols Content

Briefly, 200 μL of blueberry methanol extract, 200 μL of folinol reagent, and 1 mL of 10% sodium carbonate solution were mixed for 30 min, and the folinol method [7] was used to determine the light absorption value at 765 nm. A hydrochloric methanol solution was used as a blank, and gallic acid was used as a standard.

#### 2.10.8. Flavonoids Content

Flavonoid content was measured according to the method of Yu et al. [21] with minor modifications. Briefly, 200 μL of blueberry methanol extract was mixed with 200 μL of 5% sodium nitrite solution; 200 μL of 10% aluminum nitrate solution was added 5 min later; 1 mL of 0.5 mol/L sodium hydroxide solution was added 6 min later; and the light absorption value was measured at 510 nm after standing for 20 min. A hydrochloric methanol solution was used as a blank, and rutin was used as a standard.

### 2.11. Metabolite Analysis

#### 2.11.1. Sample Pretreatment

Blueberries were soaked in ultra-pure water for 30 s, rubbed off the coatings, dried with a napkin, homogenized, and freeze-dried. And 100 mg of blueberry powder was mixed thoroughly with 10 mL of 70% methanol solution and then placed in an ultrasonic cleaning machine (S10H, Zhiwei (Xiamen) Instrument Co., Ltd., Xiamen, China) at 900 W for 30 min. Thereafter, the sample was placed at 4 °C for 12 h, centrifuged at 12,000 rpm at 4 °C for 15 min, and 200 μL of the supernatant was taken into the special injection bottle for ultra-high performance liquid chromatography and ion mobility quadrupole time-of-flight mass spectrometry (UPLC I-class and ion IMS QTOF MS^E^, Shanghai Waters Technology Co., Ltd., Shanghai, China). Blueberry samples on day 0 were labeled as NC group, and blueberry samples on day 9 were labeled as 9-L_SP_, 9-L_82_, 9-L_46_, 9-H_sp_, 9-H_82_, and 9-H_46_, respectively. And the samples on day 15 were labeled in a similar way.

#### 2.11.2. UPLC I-Class and Ion IMS QTOF MS^E^ Condition

UPLC I-class and ion IMS QTOF MS^E^ was used on a BEH C18 column (1.7 um, 2.1 ∗ 100 mm, with pre-column). The column temperature was 45 °C, and the liquid flow rate was 0.4 mL/min. Mobile phase A was 0.1% formic acid solution, mobile phase B was 0.1% formic acid-acetonitrile solution (formic acid: acetonitrile = 1:1), and needle washing liquid was 90% acetonitrile solution. The gradient elution procedure was performed 6 times, with the initial value of 95%A + 5%B; after 3 min, it was 80%A + 20%B; after 10 min, it was 100%B; After 12 min, it was 100%B; after 15 min, it was 95%A + 5%B; after 20 min, it was 95%A + 5%B. The acquisition mode was MSE (low energy/high energy switching scanning), and the ion mode was electrospray positive and negative ion scanning, respectively. The capillary voltage was ±2 KV, and the cone hole voltage was 40 V. The atomizing air temperature was 450 °C, the atomizing gas flow rate was 900 L/h, the cone hole back blowing rate was 50 L/h, the ion source temperature was 115 °C, the scanning range was 50 to 1000 *m*/*z*, the scanning speed was 0.2 s, and the collision energy was 6 eV/20~45 eV. The online lock-in mass (online correction) was 250 pg/uL leucine enkephalin, the flow rate was 10 μL/min, the collection interval was 0.5 s, the collection time was 0.5 s, and the impact energy was 6 eV.

#### 2.11.3. Metabonomics and Bioinformatics Analysis

The original data collected by UPLC I-class and ion IMS QTOF MS^E^ were used for peak extraction, peak alignment, data normalization, and deconvolution in Progenesis QI v2.3 (Shanghai Water Technology Co., Ltd., Shanghai, China). The obtained data were matched with HMDB (www.hmdb.ca/, accessed on 6 December 2022) and Lipidmaps (https://www.lipidmaps.org/, accessed on 6 December 2022) for compound score, molecular formula, molecular mass, retention time, quality error (mDa), isotope, theory of isotope distribution similarity, ion fragment score, and other information. Compounds with the highest ion fragment score and compound score in the same isomer were selected for subsequent analysis and processing. The MetabAnalyst website (http://www.metaboanalyst.ca/, accessed on 6 December 2022) was applied for heatmap and other data analysis, and differential metabolites (DEMs) were further screened. Metabolic pathway analysis was performed using the KEGG website (http://www.kegg.jp/, accessed on 6 December 2022).

### 2.12. Data Processing and Statistics Analysis

Unless otherwise instructed, each experiment was repeated 3 times. Data were expressed in the form of the mean ± standard deviation. One-way analysis of variance (ANOVA) was applied for the analysis of variance (IBM SPSS Statistics 24 software, IBM Corporation, Armonk, NY, USA), and the Tukey method was used to perform multiple comparisons of mean differences. A *p*-value ≤ 0.05 was considered a significant difference.

## 3. Results and Discussion

### 3.1. Effect of Surfactant Formulation on Properties of Coating Solution

**Figure 1:**
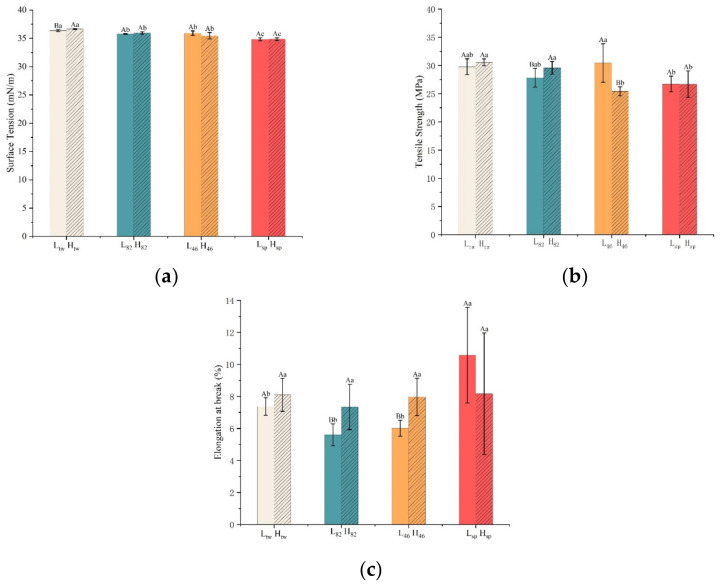
Effect of surfactant formulation on surface tension of sodium alginate coating solution (**a**), effect of surfactant formulation on tensile strength of sodium alginate film (**b**), effect of surfactant formulation on elongation at break of sodium alginate film (**c**). Different lowercase letters indicate significant differences between different surfactants of the same sodium alginate, and different uppercase letters indicate significant differences between different surfactants of the same sodium alginate species (*p* < 0.05).

It was reported that a smaller surface tension of the coating could lead to better spreadability and wettability on food surfaces, and thus the adhesion and uniformity of the resulting film were improved [22]. When the surfactant was added to the coating solution, the hydrophilic groups tended to enter the aqueous phase while the lipophilic groups enriched the surface of the system, resulting in a decrease in the surface tension. It can be seen from Figure 1a that the surface tension showed a significant downward trend when the HLB value decreased from 16.70 to 4.30. And the addition of hydrophobic Span 80 caused the surface tension to drop more obviously [23]. Besides, the surface tension of H_tw_ was higher than that of L_tw_, leading to a relative difficulty for fruit coating, and the result was in accordance with the study of Soradech et al. [24].

Table 1 presents the mean particle size of different coating solutions. On the whole, we could see that the mean particle size of group L was larger than that of group H, which might be attributed to the fact that the interactions among particles were stronger in the high-viscosity coating solution and the average distances among particles became smaller, leading to increases in the interfacial area and thus decreases in the particle size [25]. In group L, there was no significant difference in the mean particle size of the solution at each surfactant ratio, while the smallest mean particle size was obtained for the H_sp_ solution, and the value was 38.32 ± 1.25 μm. It might be attributed to the increased rigidity, enhanced stability, and reduced coalescence of the growing particles [26]. In a word, the lower HLB value with a higher proportion of Span 80 help to obtain a smaller surface tension and a lower mean particle size of the coating solution.

### 3.2. Effect of Surfactant Formulation on Properties of the Resulted Film

WVP determines whether the coated food products are easily penetrated by water, and it should be as low as possible [27]. Table 1 illustrates that the WVP of the film reached the minimum value when only Span 80 was added. The result was likely related to the fact that the hydrophobic groups of Span 80 accumulated on the surface of the film and hindered the film’s ability to absorb water from the environment. Meanwhile, as a lipophilic surfactant, the oil phase of Span 80 increased the tortuosity of water transfer in the matrix, thereby increasing the diffusion distance of water molecules in the film [28]. It was seen that the coating solution incorporated with Span 80 had a smaller mean particle size (Table 1), and thus the tortuosity factor was enhanced. Peng et al. [29] reported a higher WVP for chitosan film with the addition of Tween 80 and Span 80. Wang et al. [30] showed that with the increase in Span 80 concentration, the WVP of Konjac glycan–zein composite film first decreased and then increased. As Span 80 could not be uniformly dispersed on the film surface, the increased addition of Span 80 resulted in a decrease in the regularity and density of the network structure, making it easier for water molecules to penetrate. Table 1 also illustrated that sodium alginate with different viscosities presented different WVP, and with the increase in the proportion of Span 80, the WVP value descended from 3.17 ± 0.06 to 1.87 ± 0.10 g·mm/(m^2^·d·Pa) · 10^−6^ for group L and from 2.90 ± 0.06 to 2.43 ± 0.01 g·mm/(m^2^·d·Pa) · 10^−6^ for group H. In summary, the types and concentrations of surfactants, as well as substrate properties, could strongly affect the WVP of edible films.

Figure 1b,c presents the influence of surfactant formulation on the mechanical properties of sodium alginate film. As a whole, the TS of sodium alginate film decreased as the proportion of Span 80 increased, and the values of H_tw_ and L_tw_ were 26.72 MPa and 26.68 MPa, respectively, which was consistent with the study of Rodríguez [22], indicating that films with more Span 80 were slightly weaker. The overall EAB decreased first and then increased with the addition of Span 80, indicating that Span 80 was beneficial to the extension of sodium alginate molecules and improved the film’s flexibility. A possible explanation was that Span 80 was a small molecule that could stay in the sodium alginate molecules and function as a plasticizer; that is, it decreased the TS and increased the EAB [15,22]. It was also found that a high proportion of Tween 20 could maintain the TS of the film without reducing the EAB, inferring that a high HLB value helps to maintain the mechanical properties.

### 3.3. Effect of Surfactant Formulation on Microstructure of the Film

**Figure 2:**
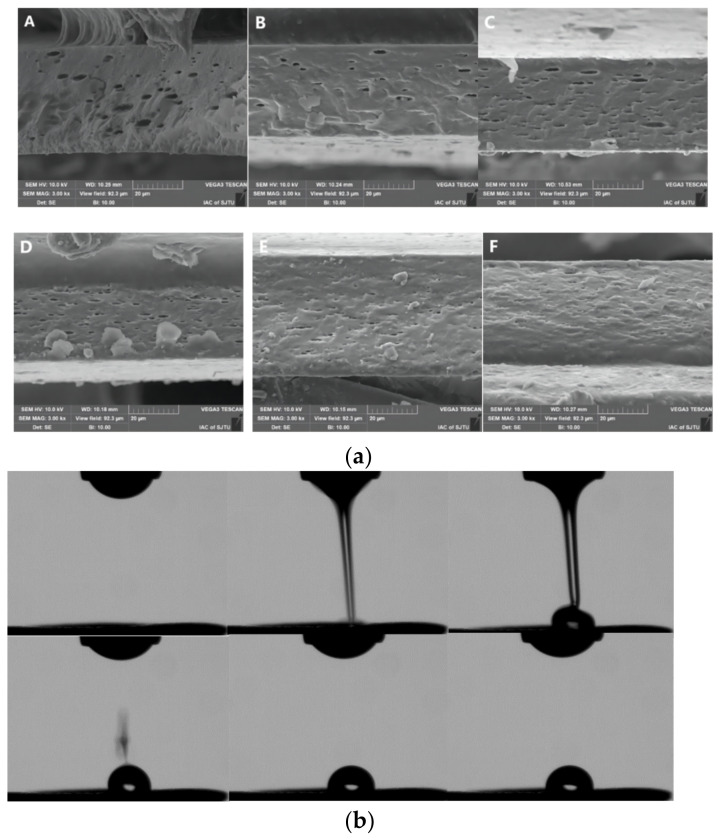

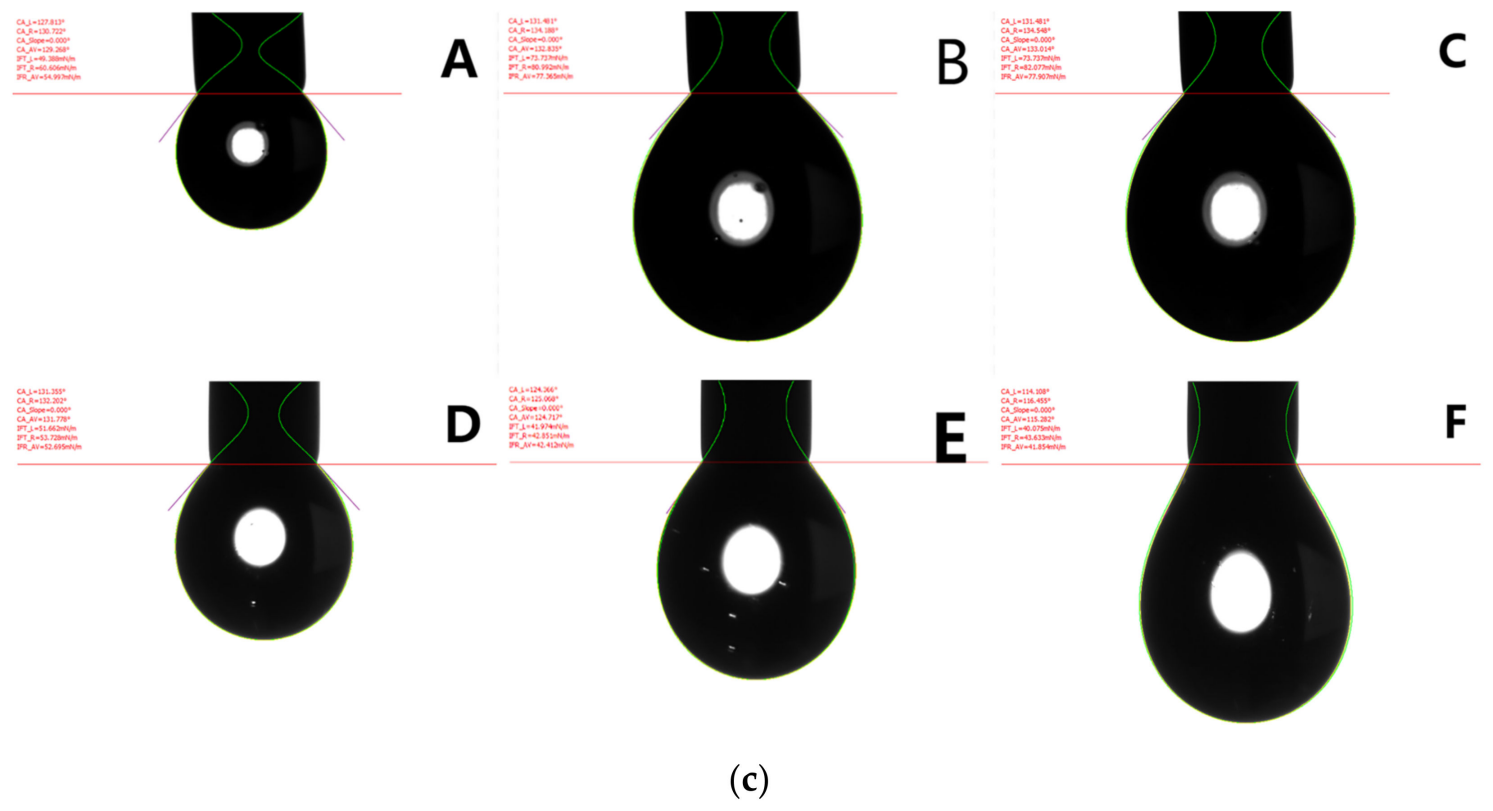
Effect of surfactant formulation on micro-structure of sodium alginate film (**A**: L_sp_; **B**: L_46_; **C**: L_82_; **D**: H_sp_; **E**: H_46_; **F**: H_82_) (**a**), injection diagram of contact angle measuring instrument (**b**), diagram of dynamic surface tension measuring using pendent drop method (**c**).

In order to study the effects of surfactant formulations with different HLB values on the microstructure of film, groups L_82_, L_46_, L_sp_, H_82_, H_46_, and H_sp_ were selected, and the SEM observations are shown in Figure 2a. For group L and group H, the section aperture formed by adding only Span 80 was the largest, while other films had lower pore numbers and pore sizes, which provided visual proof that the addition of Span reduced the TS in Figure 1b. The result might be explained by the fact that the water-insoluble Span 80 migrated to the surface of the film, varying the dispersion degree of the molecules and resulting in the film surface protruding. Subsequently, the cross-section became loosened and accompanied by pores during the film-forming process [15,30]. The film structure presented a more homogeneous and smoother surface as the content of Tween 20 increased, implying that the addition of Tween increased the stability of the oil in the film, which was consistent with previous studies [22]. By observing the pores between sodium alginate films with different viscosities, what stood out was that the pores in group H were apparently smaller than those in group L, which explained why the change in film performance for group H was generally smaller than that for group L.

### 3.4. Effect of Surfactant Formulation on Wettability of Coating Solution on Blueberry Skin

The preservation effect of the coating is impacted by its wettability on the surface of fruits and vegetables as well, and the coating must be evenly spread on the product surface to form a network with sufficient adhesion, cohesion, and durability [31]. The influence of surfactant formulation on the wettability of coating solutions was characterized by dynamic surface tension and contact angle, as shown in Figure 2b. It could be observed from Figure 2c that there were very small spots in solution droplets, namely surfactant droplets. The real-time surface tension calculation results showed that the surface tension of L_sp_ and L_82_ droplets at initial (A and D) was 54.99 mN/m and 52.49 mN/m, respectively, with no significant difference. However, the surface tension changed to 77.36 mN/m and 42.41 mN/m over suspension time (B and E). The surface tension calculated by the Asa method depended on the shape of the droplet; as hydrophobic Span 80 tended to separate from the liquid droplet and migrate upwards, the droplet surface morphology changed greatly, and the surface tension increased with time. However, the addition of hydrophilic Tween 20 effectively maintained Span 80 in the droplet and then maintained a low surface tension. The surface tension values of the L_sp_ and L_82_ groups were 77.91 mN/m and 41.85 mN/m (C and F), respectively, when the droplet reached equilibrium, showing significant differences. In the present study, surface tension values measured by the Asa method (Figure 1) differed greatly from the values measured by the platinum plate method (Table 1), which could be attributed to the stratification of surfactants mentioned above. The composite surfactant was easier to evenly distribute in the droplets and could more effectively improve the wettability.

The contact angle of the sodium alginate coating solution on the blueberry epidermis was calculated according to the Asha algorithm, and the results are shown in Table 1. The contact angle represents the wettability and spreading ability of the droplet on the material [32], and a smaller value means better wettability. For group L, the contact angle of L_sp_ was 80.00°, which was 20% higher than those of the other two groups, indicating that the addition of Tween 20 could enhance the wettability. One explanation was that Tween 20 made Span 80 more stable in the system, reducing the delamination effect and maintaining low surface tension for the bottom of the droplet (Figure 1a). It was also proven that the suspended drop method was more conducive to reflecting the real situation of wetting behavior. Moreover, the differences in contact angles among H groups were not significant, implying that high viscosity also reduced the separation ability of hydrophobic surfactants. Wiacek et al. [33] investigated the wettability of polysaccharide solutions by plasma modification and found that plasma-based surface treatment was an outstanding alternative, allowing targeted surface modification without altering material performance.

### 3.5. Effect of Surfactant Formulation on Physical Property of Blueberry

The weight loss of blueberries during storage is mainly caused by water migration and reflects the change in fruit quality to some extent [34]. It was apparent from Table 2 that with the extension of storage time, the weight loss of blueberries continually increased, which could be explained by the rapid respiration rate, continuous transpiration, dehydration, rotting of fresh blueberries, etc. [35]. The surfactants had significant impacts on weight loss, and there were larger differences in group L coated with different HLB values. The L_sp_ group coated with low an HLB surfactant formulation could better maintain the water content, which was because the lipophilicity of Span 80 could better inhibit the water penetration. The intra-group differences in group H were small, which was similar to the phenomenon in microstructure observation.

Firmness is an important economic trait and the key factor affecting the final quality of fresh blueberries. In general, firmer fruit could better withstand mechanical harvesting and subsequent transport. It could be seen from Table 2 that the firmness of blueberries basically declined first and then rose with the increase in storage time, reaching the minimum on day 3, indicating that they underwent a softening process at first and then shrank. The softening of blueberries was associated with the degradation of cell wall components such as pectin, cellulose, and hemicellulose, which might affect the mechanical properties, reduce the adhesion of cells, and cause the softening of blueberries [36]. Subsequently, the blueberries become stiffer as they shrivel from water loss. The changes were generally consistent among groups and tended to be the same on day 15. The effect of different surfactants on firmness was not obvious.

Chromatic aberration ΔE represents the sum of changes in each color axis of fruits during storage, indirectly reflecting the postharvest metabolism of fruits. The greater the ΔE, the greater the quality changes during storage. In Table 2, we can see that ΔE did not change much in the first 6 days, but the value has increased sharply since day 9. On day 15, the ΔE values decreased. Similarly, there was no significant effect of surfactants on blueberry color.

### 3.6. Effects of Surfactant Formulation on Chemical Composition of Blueberry

**Figure 3:**
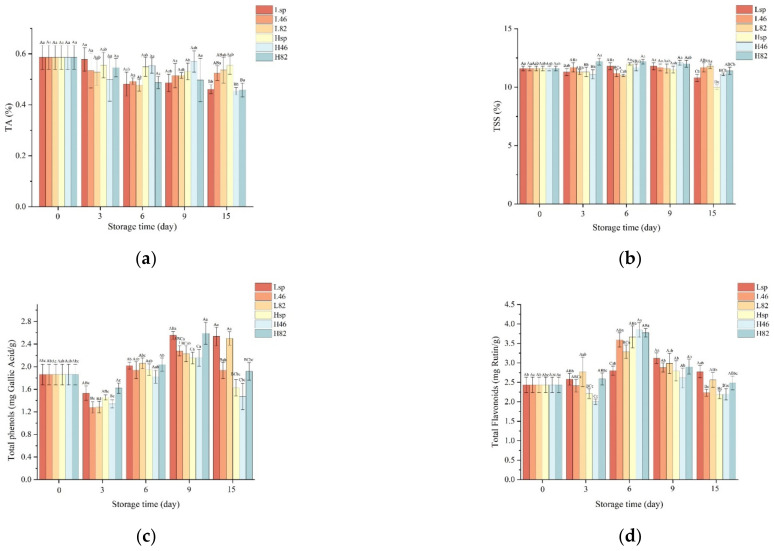
Effect of surfactant formulation on titratable acidity of blueberries (**a**), effect of surfactant formulation on total soluble solids of blueberries (**b**), effect of surfactant formulation on total phenol content of blueberries (**c**), effect of surfactant formulation on flavonoid content of blueberries (**d**). Different lowercase letters indicate significant differences between the same treatment at different storage times, while different uppercase letters indicate significant differences between different treatments at the same storage time (*p* < 0.05).

From Figure 3a, it can be seen that the titratable acidity content of blueberries in each group decreased in the early stages, and the decrease in group L was more obvious, which was related to the respiration of blueberries in the tricarboxylic acid cycle and the deamination of amino acids [23]. Similar findings were reported by Tumbarski et al. [37], and a decrease in TA was connected with the ripening and post-harvest processes of blueberries. From day 9, the content of titrable acid increased, and it might have something to do with the action of microorganisms and the accumulation of high CO_2_, resulting in rancidity [38]. As can be seen from Figure 3b, for the two different sodium alginate materials, TSS in L_sp_ and H_sp_ with low HLB decreased more rapidly, and other groups could effectively maintain the TSS content, indicating that the surfactant with low HLB value was not conducive to the maintenance of fruit sweetness, which might be related to its poor wettability.

Phenols and flavonoids are important active components in blueberries, which have antioxidant, anti-inflammatory, and anticancer functions. According to Figure 3c, at the beginning of storage, the total phenol content of blueberries decreased significantly, and this was possible due to the action between phenolic compounds and polyphenol oxidase (PPO) [39]. In the middle storage period, the total phenol content rebounded, which might be caused by the changes in non-covalent bonds between polyphenols and other macromolecules, promoting the release of phenolic substances from the bound state [40]. The observed increasing trend in total phenol content was also found in another study. According to Kraśniewska et al. [41], the total phenol content in control and coated blueberries stored at 4 °C and 16 °C increased. Alternatively, the decrease in sucrose and organic acid content during storage might provide a substrate for the synthesis of phenolic substances. In the later period, the generated phenols oxidized, and the content decreased again. Furthermore, the decline degree of phenols in group H was higher than that in group L, indicating the low-viscosity sodium alginate coating could reduce the oxidative consumption of phenols more obviously. The content of flavonoids increased first and then decreased (Figure 3d), which was consistent with the study of Zhou et al. [1]. On day 6, the content of flavonoids reached its maximum in each group, which was more abundant in the L_46_ and L_82_ groups. It reflected that sodium alginate coatings with formulated surfactants could promote the generation and accumulation of flavonoids, maintain the antioxidant capacity and physiological activity of blueberries, and thus help prolong the shelf life. Compared with group H, the changes in flavonoids in group L were relatively mild. It was speculated that sodium alginate with high viscosity resulted in high weight loss (Table 2), bringing stronger environmental pressure to blueberries and thus stimulating the metabolism related to phenols and flavonoids more strongly during storage.

### 3.7. Metabonomics Analysis

#### 3.7.1. Data Quality Evaluation

**Figure 4:**
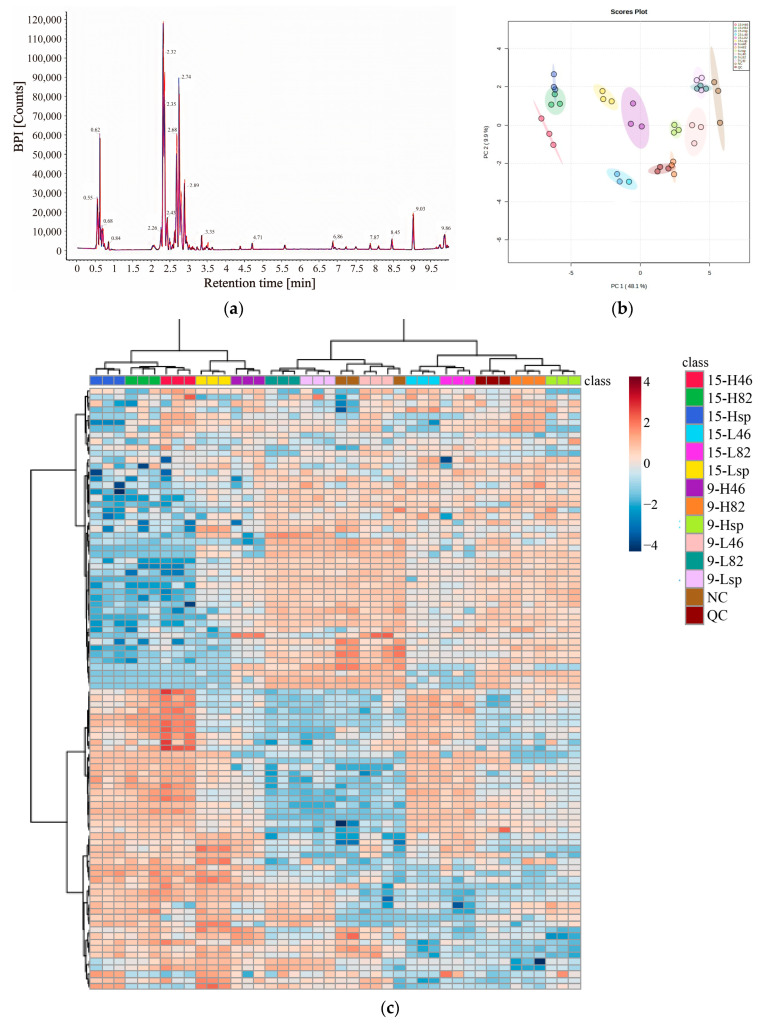
Total ion current of QC samples (**a**), principal component analysis of total samples (**b**), heat map of 96 potential DEMs (**c**).

The total ion currents of all quality control (QC) samples were overlapped and compared, and the results are presented in Figure 4a. It was observed that the response intensity of different QC samples was highly steady within the same retention time, and the size, shape, and area of the peaks were highly overlapped, indicating that the errors caused by instruments and other external factors were small and the obtained data could be further analyzed.

PCA is a widely used multivariate analysis method for data dimension reduction, data comparative analysis, and feature extraction. It was seen from Figure 4b that the top five principal components with the highest variation accounted for 74.6% of the total variation, among which PC1 = 48.1% and PC2 = 9.9%. The distances among blueberry samples in the same experimental group were close, forming tight aggregation areas. This indicated that the data in the same experimental group had good repeatability.

#### 3.7.2. Screening and Bioinformatics Analysis of DEMs

Data were imported into the MetabAnalyst website to obtain each metabolite variable importance for the projection (VIP) and metabolite difference analysis value *p*. Under the conditions of VIP ≥ 1, *p* ≤ 0.05, 96 potential DEMs were screened. All potential DEMs were analyzed using heat maps, and the results are shown in Figure 4c. The 96 potential DEMs could be divided into two groups. The first type was the metabolites whose content was up-regulated over time, including galactose, UDp-D-xylose, sorbitol, quercetin, etc. The second was metabolites whose levels declined over time, including lactose, naringin, and phosphate esters. Studies have shown that sorbitol is positively correlated with fruit water loss, so fruits have stress resistance in harsh environments [42]. It can be inferred that sodium alginate coatings can play a role in the preservation of blueberries by regulating the metabolic pathway of sorbitol. According to Figure 4c, on day 15, the H_46_, H_82_, L_46_, and L_82_ groups reported significantly more ketones and alcohols than H_sp_ and L_sp_, such as 11-methylgerberinol, 8-hydroxyondansetron, 3 alpha-hydroxyoreadone, pterosin N, 2,2-dichloro-1,1-ethanediol, eremopetasidione, and so on. These substances were related to the stress resistance and antioxidant properties of fruits, inferring that formulated surfactants could have a better preservation effect, which was correlated to the chemical compound changes. The cluster analysis in Figure 4c showed that 9-L_46_ had the most similar properties to the NC group, and the quality of group L was better than that of group H as a whole.

**Figure 5:**
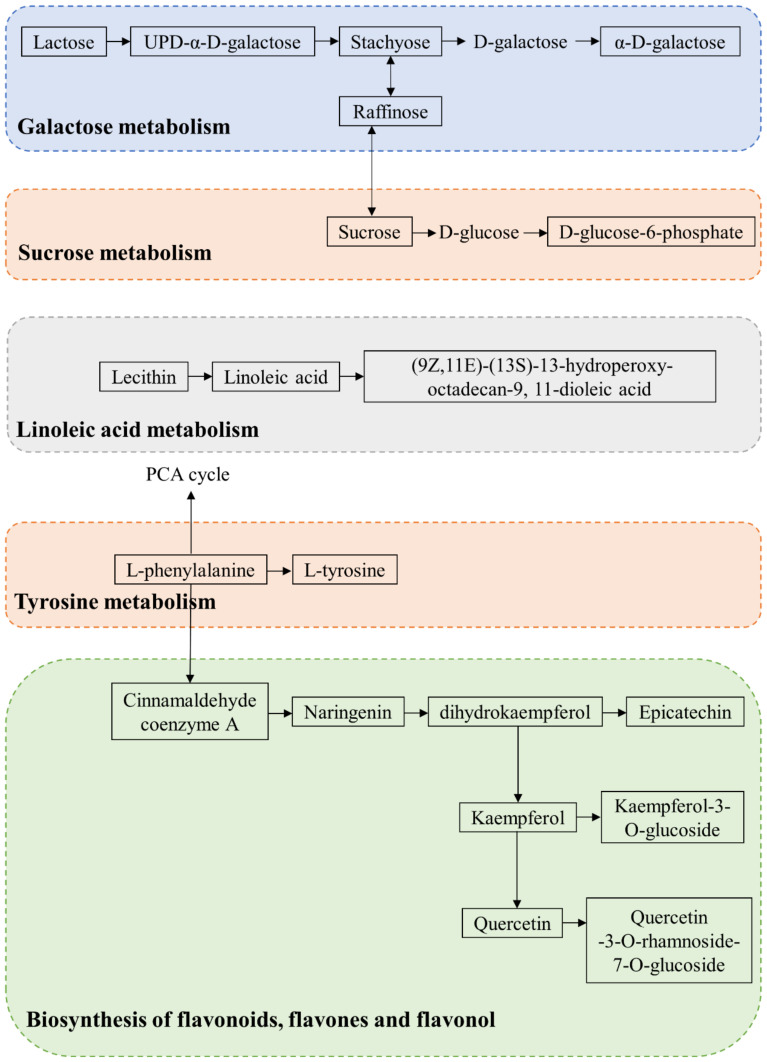
Metabolic pathway of blueberries during storage. The substances with black frame in the figure are the differential metabolites screened in the earlier stage, and the substances without black frame are the original metabolites in the metabolic map.

In order to further explore the metabolic process of blueberries, 96 potential differential substances were introduced into MetabAnalyst for metabolic pathway analysis, and finally, 31 substances with KEGG coding were obtained, including amino acids, sugars, linoleic acid, and flavonoids. The corresponding metabolic pathway diagram is shown in Figure 5. The carbohydrate metabolism in blueberries included lactose, galactose, hydrothreose, raffinose, sucrose, glucose, etc., which were closely related to the energy metabolism, hardness, and soluble solid content of blueberries. The sucrose contents of blueberries on days 9 and 15 were higher than those on day 0, which was also a sign that blueberries tended to mature. Raffinose was an important metabolite in response to a low-temperature environment. The content of raffinose in group L was lower than that in group H, suggesting that the low-viscosity sodium alginate coating was more helpful for blueberries to resist low temperatures. The metabolism of linoleic acid has a complete pathway in blueberry metabolism, which is a part of secondary metabolism in plants and is of importance in plant disease resistance [43]. (9Z,11E)-(13S)-13-hydroperoxy-octadecan-9,11-dioleic acid was the final metabolite of the 1,2-diacyl-sn-glycero-3-phosphocholine, which was less in group L, so it might have a better preservation effect. Tyrosine metabolism is a key pathway connecting the primary metabolism and secondary metabolism of individual fruits [44]. L-phenylalanine and L-tyrosine involved in this metabolic pathway showed no significant difference between the NC group and the coated group on the 9th day, which could be attributed to the good preservation effect of sodium alginate coating. Flavonoids such as naringin, kaempferol, and quercetin are beneficial to plant growth as physiologically active compounds, stress protectants, attractants, and ingestion deterrence. Flavonoids not only improve the antioxidant capacity of fruits to a large extent but also play a positive role in their resistance to pathogens and fungal decay after harvest [45]. According to the present analysis, blueberries significantly enhanced their ability to withstand biological and abiotic stresses by activating the biosynthesis of flavonoids, among which the contents of flavonoids in 15-H_sp_, 15-H_82_, and 15-H_46_ were significantly higher. It was also found that the preservation effect of sodium alginate coatings with high viscosity was lower than that of the low viscosity group.

## 4. Conclusions

This study was set out to explore the effect of Tween 20/Span 80 surfactant formulations on the film-forming property, wettability, and preservation performance of electrostatical sprayed sodium alginate films. The study has identified that a higher proportion of Tween 20 could improve the uniformity, reduce the surface tension, ensure good wettability of the coating solution, and maintain an excellent TS without reducing the EAB of the film. On the other hand, the addition of Span 80 could reduce the mean particle size of the coating solution and improve the water-resistance performance of the film. The blueberry preservation experiment confirmed that low HLB coating was helpful for maintaining blueberry weight and firmness but was not conducive to the maintenance of sweetness. Metabonomics analysis showed that the regulation of the postharvest metabolism of blueberries by coating involved amino acid, carbohydrate, flavonoid, and linoleic acid pathways. Sodium alginate coating with medium HLB had both good film-forming ability and wettability and was more conducive to the preservation role. In summary, except for reducing the surface tension and improving the wettability of coating solutions, the distribution of surfactants is also of great importance, which is related to the HLB value. Thus, the right choice of the HLB value was crucial for the preservation effect of edible coatings and needed to be accurately designed when determining the film-forming formula.

## Figures and Tables

**Table 1:** Effect of surfactant formulation on the mean particle size, WVP, dynamic surface tension, and contact angle.

Group	D_43_/[μm]	WVP /[g·mm/(m^2^·d·Pa) · 10^−6^]	Dynamic Surface Tension/[mN/m]	Contact Angle/[°]
L_tw_	129.96 ± 13.09 ^Aa^	3.17 ± 0.06 ^Aa^	-	-
L_82_	129.90 ± 4.72 ^Aa^	3.15 ± 0.12 ^Aa^	41.85	66.53 ± 1.86 ^cd^
L_46_	131.26 ± 14.56 ^Aa^	2.71 ± 0.26 ^Ab^	42.30	62.75 ± 2.20 ^d^
L_sp_	123.31 ± 6.80 ^Aa^	1.87 ± 0.10 ^Bc^	71.32	80.00 ± 1.76 ^ab^
H_tw_	47.22 ± 1.72 ^Ba^	2.90 ± 0.06 ^Ba^	-	-
H_82_	42.24 ± 1.24 ^Bb^	2.99 ± 0.05 ^Ba^	45.52	80.97 ± 1.79 ^a^
H_46_	48.13 ± 0.84 ^Ba^	2.90 ± 0.14 ^Aa^	48.25	73.35 ± 1.73 ^abc^
H_sp_	38.32 ± 1.25 ^Bc^	2.43 ± 0.01 ^Ab^	73.75	72.42 ± 3.97 ^bc^

Different lowercase letters indicate significant differences between different surfactants of the same sodium alginate, and different uppercase letters indicate significant differences between different surfactants of the same sodium alginate species (*p* < 0.05).

**Table 2:** Effect of surfactant formulation on firmness and color of blueberries.

Index	Storage Time/Day	L_sp_	L_46_	L_82_	H_sp_	H_46_	H_82_
Weight loss/%	0	0.00 ± 0.00 ^Ac^	0.00 ± 0.00 ^Ad^	0.00 ± 0.00 ^Ac^	0.00 ± 0.00 ^Ae^	0.00 ± 0.00 ^Ae^	0.00 ± 0.00 ^Ad^
	3	0.27 ± 0.04 ^Cc^	0.53 ± 0.02 ^Bd^	0.47 ± 0.05 ^Bc^	0.69 ± 0.11 ^Ad^	0.41 ± 0.07 ^Bd^	0.24 ± 0.03 ^Cd^
	6	1.21 ± 0.14 ^Cb^	2.17 ± 0.09 ^Ac^	1.87 ± 0.02 ^Bbc^	2.41 ± 0.16 ^Ac^	1.78 ± 0.10 ^Bc^	1.60 ± 0.08 ^Bc^
	9	1.70 ± 0.05 ^Db^	3.78 ± 0.01 ^ABb^	3.15 ± 0.25 ^BCab^	3.91 ± 0.28 ^Ab^	3.57 ± 0.14 ^Cb^	3.45 ± 0.52 ^ABb^
	15	3.52 ± 0.56 ^Ca^	7.42 ± 0.64 ^Aa^	5.13 ± 1.70 ^BCa^	5.54 ± 0.34 ^ABCa^	5.16 ± 0.11 ^BCa^	5.78 ± 0.05 ^ABa^
Firmness /N	0	20.33 ± 0.80 ^Ab^	20.33 ± 0.80 ^Ab^	20.33 ± 0.80 ^Ab^	20.33 ± 0.80 ^Ac^	20.33 ± 0.80 ^Ab^	20.33 ± 0.80 ^Ac^
	3	17.11 ± 0.51 ^Cc^	17.55 ± 0.85 ^BCc^	18.69 ± 1.02 ^Bc^	21.92 ± 0.60 ^Ab^	18.31 ± 0.69 ^Bc^	18.32 ± 1.11 ^Bd^
	6	22.32 ± 1.63 ^Aa^	19.90 ± 0.97 ^Bb^	21.49 ± 1.46 ^ABab^	21.90 ± 1.56 ^Ab^	22.17 ± 0.75 ^Aa^	22.24 ± 1.05 ^Ab^
	9	22.81 ± 1.86 ^Aa^	19.79 ± 1.78 ^Bb^	20.94 ± 1.30 ^ABab^	21.39 ± 0.84 ^ABbc^	20.95 ± 0.43 ^ABb^	21.93 ± 1.64 ^Ab^
	15	23.30 ± 0.76 ^BCa^	24.63 ± 0.55 ^Aa^	22.43 ± 1.36 ^Ca^	23.51 ± 0.90 ^ABCa^	23.12 ± 1.10 ^BCa^	24.22 ± 0.82 ^ABa^
ΔE	0	0.00 ± 0.00 ^Ad^	0.00 ± 0.00 ^Ad^	0.00 ± 0.00 ^Ad^	0.00 ± 0.00 ^Ad^	0.00 ± 0.00 ^Ad^	0.00 ± 0.00 ^Ad^
	3	0.82 ± 0.37 ^Bc^	0.85 ± 0.47 ^Bc^	0.86 ± 0.51 ^Ac^	0.98 ± 0.62 ^Bc^	0.77 ± 0.46 ^Bc^	1.38 ± 0.63 ^Bc^
	6	0.96 ± 0.53 ^BCc^	0.78 ± 0.45 ^Cc^	0.92 ± 0.70 ^BCc^	1.23 ± 0.68 ^ABc^	0.90 ± 0.46 ^BCc^	1.35 ± 0.73 ^Ac^
	9	2.85 ± 0.91 ^Da^	3.76 ± 0.51 ^BCa^	4.01 ± 0.73 ^Ba^	4.61 ± 0.57 ^Aa^	2.27 ± 0.68 ^Ea^	3.42 ± 1.16 ^Ca^
	15	1.63 ± 0.55 ^ABCb^	1.40 ± 0.74 ^BCb^	1.25 ± 0.58 ^Cb^	1.92 ± 0.97 ^Ab^	1.72 ± 1.03 ^ABCb^	1.83 ± 0.97 ^ABb^

Different lowercase letters indicate significant difference between the same treatment at different storage times, while different uppercase letters indicate significant difference between different treatments at the same storage time (*p* < 0.05).

## Data Availability

The data presented in this study are available on request from the corresponding author.
